# Ligand Access Channels in Cytochrome P450 Enzymes: A Review

**DOI:** 10.3390/ijms19061617

**Published:** 2018-05-30

**Authors:** Philippe Urban, Thomas Lautier, Denis Pompon, Gilles Truan

**Affiliations:** Laboratoire d’Ingénierie des Systèmes Biologiques et des Procédés, Université de Toulouse, CNRS, INRA, INSA, 31000 Toulouse, France; lautier@insa-toulouse.fr (T.L.); dpompn@free.fr (D.P.)

**Keywords:** P450, QSAR, channel, polycyclic, substrate specificity, CAVER, Computing Cavities, Channels, Pores and Pockets (CCCPP), Random acceleration molecular dynamics (RAMD), haloalkane dehalogenase, structure

## Abstract

Quantitative structure-activity relationships may bring invaluable information on structural elements of both enzymes and substrates that, together, govern substrate specificity. Buried active sites in cytochrome P450 enzymes are connected to the solvent by a network of channels exiting at the distal surface of the protein. This review presents different in silico tools that were developed to uncover such channels in P450 crystal structures. It also lists some of the experimental evidence that actually suggest that these predicted channels might indeed play a critical role in modulating P450 functions. Amino acid residues at the entrance of the channels may participate to a first global ligand recognition of ligands by P450 enzymes before they reach the buried active site. Moreover, different P450 enzymes show different networks of predicted channels. The plasticity of P450 structures is also important to take into account when looking at how channels might play their role.

## 1. Introduction

Cytochrome P450 (P450) have been found in most of organisms where they were looked for [1], with some noticeable exceptions, such as *E. coli.* The mechanism of action of all P450 enzymes, except a few ones, is to catalyze molecular dioxygen reduction by splitting the dioxygen molecule in two, one oxygen atom is inserted in the substrate to yield the oxidized product, and the second is reduced to water, a typical monooxygenase activity [2]. Two biochemical properties characterize P450 enzymes among other hemoproteins. The first one is that the reduced P450 binds CO. The reduced-CO complex has a Soret band with a maximum displaced at 450 nm, hence its name [3]. The second property is the presence of a strong bond between the heme iron ion and the sulfur thiolate of a cysteine residue [4]. 

The P450 enzymes, which are divided into gene families and subfamilies on the basis of the amount of identity between amino acid sequences [5], can be broadly classified in two main classes depending on their global substrate specificity. A first class includes P450 enzymes that have a strong preference for a single substrate. These P450s belong mostly to the plant and bacterial worlds [6,7], and also include P450s of the steroidogenesis and eicosanoid pathways in animals [8,9]. The second class comprises P450 enzymes featuring a broad specificity for substrates, often very different in size and chemical nature [10], mostly comprising the P450s that are involved in the drug and pollutant metabolism in vertebrates. Consequently, P450 enzymes are frequently designated as unspecific monooxygenases (EC1.14.14.1).

**Figure 1:**
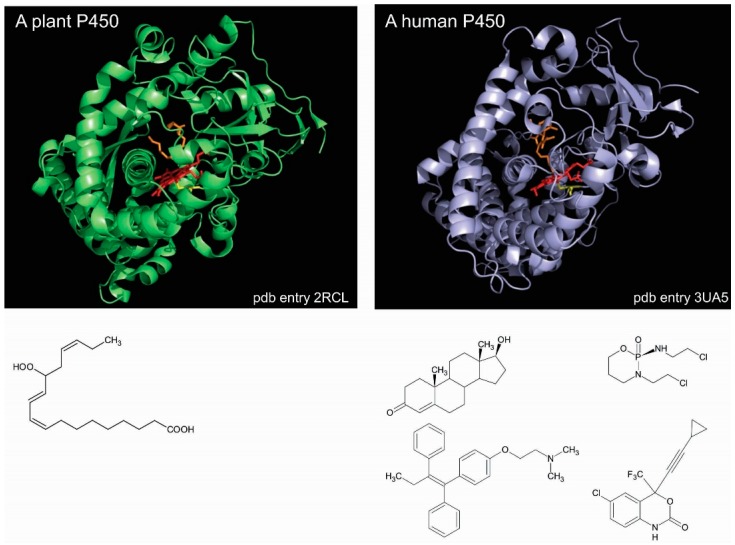
P450 enzymes exhibit similar structures. Left panel, CYP74A from *Arabidospsis thaliana* complexed by 12R,13S-vernolic acid at 2.4 Å resolution (pdb entry 3dam) [16]. Right panel, human CYP2B6 complexed by amlodipine at 2.24 Å resolution (pdb entry 4zv8) [17]. The amino acid sequence identity between the two proteins is 14%. The heme is represented in both enzymes as red sticks and the bound ligand as orange sticks. The heme-liganding cysteine is colored yellow. Protein visualizations were performed by using PyMol (Delano Scientific LLC, San Carlos, CA, USA). The caption under both of the crystal structures represents the single CYP74A substrate (allene oxide) on the left and some of many CYP2B substrates on right.

P450 enzymes of either classes share the same similar catalytic cycle [11,12] and a similar folding. The numerous crystal structures available show that P450s share a common triangular prism-like domain rich in α-helices. Such a high degree of similarity across a huge diversity of P450 sequences and functions makes it difficult to readily understand the structural bases of this large array of substrate specificities, so different from one P450 to the other [13,14]. For instance, *Arabidopsis thaliana* CYP74A, which is an untypical P450 enzyme that does require molecular dioxygen, is specific of a single substrate yielding an allene oxide [15]. This plant P450 exhibits a crystal structure that is highly similar to any other P450 enzymes, such as that of human CYP2B6, which reduces molecular dioxygen and reacts with hundreds of different substrates (Figure 1). 

The extraordinary chemical ability of P450 enzymes is mainly based on three features that all of these enzymes share. One is the fact that the active site of a P450 is a cavity that is buried within the protein molecule, and thus, clearly separated from the solvent [18]. The second is the fifth coordination bond of the heme that links a positively charged iron ion to a negatively charged thiolate sulfur atom [19]. This iron–cysteinate bond is at the heart of the different redox states that the iron ion may access during P450 catalysis, leading to the sophisticated catalytic cycle of P450 enzymes. The third is the fact that two highly synchronized electron transfer steps are required to allow for the P450 catalytic cycle to reach its productive step [20]. Besides these common features, all of the P450 enzymes also present a typical structural feature, the absence of a clear visible access between the protein surface and the active site that is always buried deep in the protein core. Hence, any potential ligand has to find a way through the body of the P450 protein. However, some crystal structures of bacterial P450s show an active site that is deep in the core protein, but with an access channel that is clearly visible.

In silico analyses of P450 crystal structures for possible access channels have systematically predicted a network of channels connecting the buried catalytic cavity to the protein distal surface [21]. It was proposed that these channels might be the pathways that were used by the different ligands to reach the active site heme iron. Differences in the networks of channels among P450 enzymes may account for the differences in ligand selectivity, and hence, for the ways that P450 enzymes control at several levels their substrate selectivity. Some residues forming the entrance of the channel or forming the wall could be implied in a primary selection of the ligand before it reaches the active site. 

A recent review provides a comprehensive presentation of the different computational methods that were developed to characterize channels in proteins and their dynamics, and how this in silico information help us to understand the influences of channels on both substrate selectivity and catalysis in enzymes [22]. Very interestingly, when applied to the CSA (Catalytic Sites Atlas) library of 4306 enzyme structures, one of the algorithms unexpectedly revealed that more than 64% of enzyme structures exhibit a channel that is at least 15 Å long going from the active site to a spot on the surface of the protein where it egresses [23]. This study thus, somewhat surprisingly, indicated that the presence of channel(s) in protein might be the norm rather than the exception.

## 2. Identifying Channels in P450 Enzymes

The observation, some fifty years ago [24,25,26], of cavities not being accessible from the solvent in the crystal structure of sperm whale myoglobin paved the way to looking at channels as a succession of linked cavities forming a continuous path inside proteins. The first algorithm devised to look at and display “cavity-like binding regions” in proteins was presented some thirty years ago by Ho and Marshall on the binding of MVT-101 inhibitor to the human immunodeficiency virus HIV-1 protease [27]. Since this first attempt, more than thirty geometric algorithms have been developed for finding cavities and channels in the crystal structures of proteins. 

For a rather long period, besides the ribosomal tunnel of the nascently synthesized polypeptide [28], cytochrome P450s and haloalkane dehalogenases were the only known enzymes with a deeply buried active site with no easily visible pathway leading from the protein surface to the catalytic cavity [29,30]. Historically, the first attempt to identify access channels in cytochrome P450 was made twenty years ago by Rebecca Wade and her coworkers on cytochrome P450cam (CYP101A1) [31]. They were using a thermal motion pathway analysis that calculates the manner by which contiguous triplets of atoms of the highest average temperature factors in a crystal structure may form a pathway from a buried cavity to the protein surface. In the same paper, they confirmed findings from this in silico thermal analysis by another search for channels by an in silico random expulsion force molecular dynamics (REMD) analyses, later renamed random acceleration molecular dynamics (RAMD), using a camphor molecule that was bound at the P450 active site. In RAMD, an additional randomly oriented force is applied to the center of mass of the ligand, which is represented in full atomic detail and flexibility. The direction of the force is changed during the simulation if, upon monitoring, the ligand displacement over a specified time window is below a defined threshold distance. The RAMD method allows for many unbiased ligand egress events to be observed in a short simulation time and provides information on egress route probabilities and egress mechanisms, such as induced fit. This computational method, as well as the steered molecular dynamics approach, identified several main access/exit channels in the crystal structure of P450cam [32,33].

Since these founding papers, other algorithms for discovering channels in protein were developed to find all the possible paths by which a given ligand accesses and exits the P450 catalytic cavity, such as accelerated molecular dynamic simulations [34]. Other methods, based on protein structure rendering by Delaunay triangulation, allow for the fast identification of channels in proteins for a ligand represented by a sphere. The most used algorithms, the CAVER-MOLE series that was developed by Michal Otyepka and his collaborators [35,36], use a fixed-size sphere of specified diameter placed close to the heme iron ion in the distal cavity of the P450 enzyme to identify all of the direct paths connecting the buried active site to the protein surface by rolling it out of the active site. Some useful web-tools are also freely available, such as MOLEonline (https://mole.upol.cz/), which calculates access channels and their physicochemical properties in any crystal structure that is provided by the user and ChannelsDB (https://webchemdev.ncbr.muni.cz/ChannelsDB/), which presents a database of all annotated access channels in enzyme structures, including cytochromes P450. This database contains information about channel positions, geometry, and physicochemical properties that are presented in a clear and interactive manner facilitating interpretation. 

Fifteen years ago, Wade et al. presented the first review of the different ligand routes that they had identified in both bacterial and mammalian P450 structures with the RAMD approach [37]. A decade ago, Cojocaru et al. have classified and named the different access channels that were found by CAVER on structurally characterized P450 enzymes in a systematic manner [38]. The different ligand access channels that were identified in P450 enzymes were each named by a different number (channels 1, 2, 3, 4, W, and S). These channels are lined by different secondary structure elements and are located in topologically different parts of the structure. For the channel 2, an additional letter (2a, 2b, etc.) refers to spatially different parts of a global route 2, all being lined at least by the B–C loop.

Very recently, another algorithm, based on alpha shapes method, seeks both all concave regions at the surface of a protein and internal channels and cavities by taking into account both the size and the shape of the ligand represented as a cylinder [39]. Since most P450 substrates are disc-shaped, the use of a flattened cylinder is more adapted to the substrate reality than a fixed-size ball. However, the computation time is increased since the object to move is not defined by a single parameter (sphere diameter) anymore, but by two parameters (cylinder diameter and cylinder height). 

Finally, one of the last developments in the computational search for channels in proteins is the introduction of memetic algorithms [40]. This article, originating from physicists from a department of Astronomy and Informatics, deals with the difficulty in modeling ligand egress pathways by ‘classical’ molecular dynamic simulations, such as RAMD, CAVER, and MOLE. Finding the right combination of parameters is not trivial and the percentage of successful dissociation events (i.e., a molecular dynamics simulation that yields a probable exit pathway) was as low as 19% in some cases, and as high as 60% with CAVER. The memetic algorithm presented, memory enhanced randoma (MERA) and molecular dynamics and immune algorithmor MERA-IA, adds a representation of memory that is equivalent to pheromone trails that are used by ants and that was mathematically simulated in Ant Colony Optimization algorithms. During simulations on protein structure, the exiting ligand leaves a “pheromone” trail used as exit information for the next simulations. Because of that, during molecular dynamics simulations in the MERA-IA algorithm, the ligand is subjected to two forces; the first one (also found in classical algorithms) is pushing it randomly outside the protein. The second force is constantly adjusted to direct the moving ligand toward the closest area of the protein with the highest concentration of ‘pheromone’ at each position that it reaches. When applied to proteins, this memetic algorithm finds successful simulations with a percentage ranging from 96 to 100% of all simulations carried out, an excellent result that demonstrates the validity of this method.

**Figure 2:**
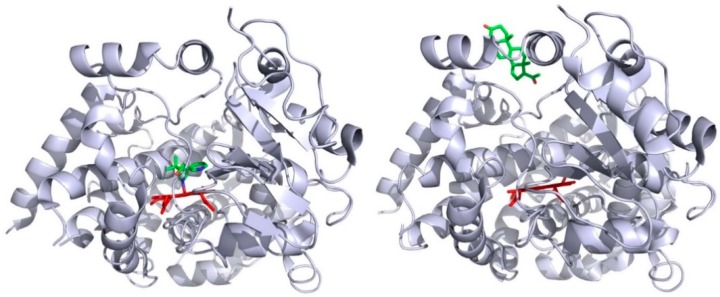
Positions of metyrapone (**left panel**) and progesterone (**right panel**) bound to CYP3A4. The crystal structures are taken from the Protein Data Bank entry 1w0f (**left panel**) and 1w0g (**right panel**) [50]. The heme is shown in red and the ligands are colored by atom type (green, carbon; red, oxygen, blue, nitrogen).

Deductions from these in silico approaches are found to be consistent with some biochemical data. P450cam (CYP101), which is one of the best characterized of all P450 enzymes, is considered as a model for P450s with narrow substrate specificity [41]. This bacterial P450 catalyzes the 5-hydroxylation reaction of camphor [42], a terpene that can be used by the bacterium *Pseudomonas putida* as its sole carbon source [43]. P450cam was considered for a long time to not show structural flexibility, since the first crystal structures of the substrate-free and substrate-bound forms of P450cam were similarly exhibiting a compact conformation [44]. But, some fifteen years later, Goodin and his collaborators showed that P450cam can visit an open conformation in the absence of substrate when the concentration in potassium ion in the crystallization medium is considerably lowered [45]. The opening of the P450cam structure is due to a concerted backward movement of the F- and G-helices and an uprising of the F/G loop, together with a higher degree of flexibility of the B/C loop [46]. This opening of P450cam structure unveils an access channel linking the protein surface, in the vicinity of the F/G loop, to the catalytic cavity. Finally, P450cam was shown to adopt up to three different conformations, named C, I, and O, each being adapted to the substrate size [47]. The C-conformation is the one of the camphor complex and with substrate-free P450cam at high potassium ion concentration. The I-conformation is observed with some adamantine-alkyl derivatives as ligands. The O-conformation is the one observed with substrate-free P450cam at low potassium ion concentration. In numerous experiments, multiple molecules of ligands have been observed to bind simultaneously to P450 enzyme. Among others, the allosteric response observed with some P450 kinetics is a good indicator of the fact that several ligand molecules can simultaneously bind to the monomeric protein [48,49]. Alternatively, some crystal P450 structures present a protein that exhibits several molecules of tightly bound ligands, as, for instance, the case of human CYP3A4 with a metyrapone molecule bound within the catalytic cavity and a progesterone molecule bound at its surface [50]. Figure 2 shows the exact location of these two ligands, as seen in the crystal structure. It appears that the inhibitor metyrapone binds the heme iron ion by one of its pyridine nitrogen, and that the progesterone is bound on the CYP3A4 surface to a pocket made by the F/G region, and which colocalized with the mouth of either CAVER channel 3 or 4 in P450 enzymes. 

This binding could not result from a crystallization artifact, but be physiologically sounding, as proposed by Davydov and Halpert [51]. Very recently, Denisov et al. have demonstrated that the drug-drug interaction existing between atorvatstatin, which is a cholesterol-lowering drug, and dronedarone, an antiarrythmic drug, on CYP3A4 was indeed taking place by a crosstalk between a site on the surface of the enzyme, at which progesterone is seen bound in the crystal structure, and the buried active site [52]. The binding of an effector molecule at the peripheral site was shown to improve or inhibit the metabolism of the active site-bound substrate by its repositioning in the catalytic cavity. 

Using ^1^H-^13^C HSQC nuclear magnetic resonance NMR titrations of bacterial P450cam (CYP101), it has been shown that, in the presence of excess camphor, a second camphor molecule binds at the surface of the protein, besides the one bound within the buried active site [53]. Among the CYP101A1 homologues, CYP101D2, which is a camphor 5-hydroxylase from *Novosphingobium aromaticivorens*, has been crystallized in both substrate-free and camphor-bound forms [54]. The camphor-bound crystal structure show additional regions of electron density that were modelled as camphor molecules bound to camphor-soaked CYP101D2 in other regions of the protein than the active site. One camphor molecule is found bound to the active site within the catalytic cavity. A second molecule was found obstructing a channel linking the active site to the protein surface close to the channel entrance near the F/G loop. The third camphor molecule was observed lying on the protein surface near the junction of the F-helix with the F/G loop. This third molecule could be at the entrance of an access channel that it closes in this structure. 

In the crystal structure of CYP102, also known as P450 BM3, a fatty acid hydroxylase P450 enzyme that is isolated from *Bacillus megaterium*, the active site cavity is accessible through a clearly visible long channel that links the heme iron distal environment to the bulk solvent. The entrance of this channel is composed of a patch of hydrophobic residues that may be important for the initial recognition of lipophilic substrates since they are solvent-exposed and mobile [55].

In total agreement with the results of NMR titrations that were performed on P450cam, a two-step recognition process was also described in ^1^H-NMR measurements on bacterial P450 BM3. Paramagnetic relaxation experiments indeed demonstrated that the reduction of the P450 BM3-laurate complex is accompanied by a structural change leading to a 6-Å movement of laurate into the correct position for hydroxylation to occur. This movement corresponds to a sliding of the molecule down the channel [56]. When applying hydrostatic pressure perturbations to bacterial CYP107 (P450eryF), it was observed by fluorescence spectroscopy that a conformation transition occurs when substrate binds and that three substrate molecules bind to the P450 [57]. Two of these three molecules are located within the catalytic cavity and the third is located at the protein surface.

All of these experiments agree with the notion that a correlation exists between the positions of the channels identified computationally and the positions in P450 crystal structures of ligands bound at sites distant from the active site.

## 3. Channel Dynamics in Cytochromes P450

Twenty years ago, when molecular dynamics simulations were calculated separately for each of the two CYP102 molecules that were seen in the asymmetric unit of the crystal structure, it was evident that the entrance of the visible access channel is more mobile and flexible in one the molecule than in the other. For CYP102 molecule 1, the width of the mouth undergoes fluctuations of about 1 Å, whereas for CYP102 molecule 2, these fluctuations reach up to 7 Å. The authors presumed that the binding of different ligands will induce the entrance of the channel to adopt different conformations from within the wide range of accessible structures [58]. In this historical study, a P450 enzyme was shown for the first time to exhibit some flexibility, coincidental with the opening at the protein surface of an access channel to its buried active site. 

**Figure 3:**
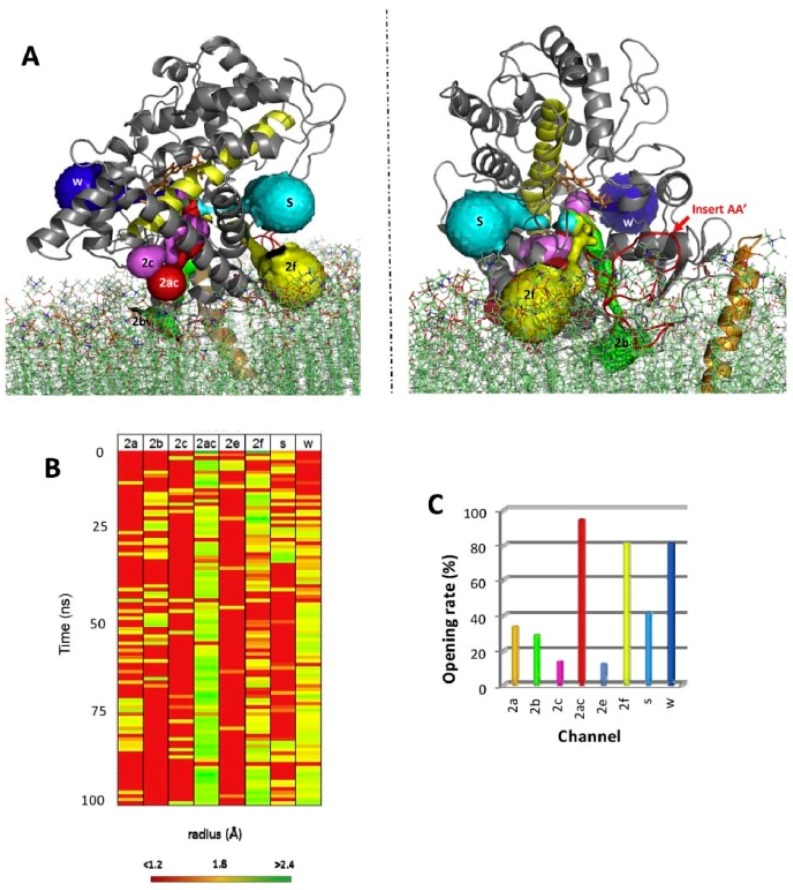
Channels dynamics in CYP2U1. (**A**) Representation of the CAVER channels labeled according to Wade et al. nomenclature [38]. The two figures are deduced from a 90° rotation. (**B**) Heatmap of the time course of opening and closing of channels during molecular dynamics simulations the color map ranges from red to yellow for closed channels (radius < 1.2 Å) and from yellow to green for open channels (radius > 2.4 Å). (**C**) The percentage of trajectory frames in which the identified channels are open (with a radius > 1.2 Å) is shown with the same color code used for channel as in panel A. Clearly, channel 2ac is the most frequently opened in this molecular dynamics simulation of CYP2U1. This illustration comes from Ducassou et al. [60].

For all mammalian P450 enzymes that were considered, a network of several access channels linking the catalytic cavity to the distal face of the protein is consistently predicted. To our knowledge, no plant P450 enzyme has been analyzed for CAVER channels. Access channel could be opened or closed only by the movement of side chains thus maintaining the topology of the protein as seen in the crystal structure, as is systematically predicted by molecular dynamics simulations (Figure 3). However, in P450 enzymes, discrete side chains reorganizations and some secondary elements are also prone to conformational change, both types of protein dynamics seem to take place [59].

Detailed stopped-flow kinetics of binding of camphor to cytochrome P450cam have been studied for site-directed mutants of P450cam. It was shown that one of the mutants, Thr192Glu, was presenting altered energetics of camphor association relative to the wild-type enzyme, as observed in the Arrhenius plot of its binding kinetics. Threonine 192 resides on the F/G loop and belongs to the mouth of the putative ligand access channel of the enzyme. This result suggests that in P450 enzymes preliminary ligand recognition takes place at the surface of the enzyme before channeling it to the catalytic cavity [61]. 

Besides the potential role of structural elements on the P450 molecule, another important point to consider is the fact that all P450s in eukaryotes are membrane bound. Some thirty years ago, a study on hepatic microsomal fractions showed that the ethoxyphenoxazone deethylase activity from rat liver microsomal fractions was found to decrease with time, but the addition of synthetic phospholipids was restoring the activity [62]. This was the first study to demonstrate that phospholipids were not just playing a structural role bymaking the membrane, but also could play a role in the function of the P450 enzymes. It is known, though surprisingly enough not much worked, that phospholipids indeed play a role in modulating the activity of membrane-bound P450 enzymes [63,64,65].

A recent study has also shown in silico that the phospholipids may modulate the dynamics of access channels in human CYP1A2 [66]. One of the results presented shows that, during molecular dynamics simulations, a molecule of 1,2-dilauroyl-*sn*-glycero-3-phosphocholine (DLPC) can penetrate in one of the CYP1A2 access channels (namely tunnel 2d). The penetration progress, monitored as the time evolution of distance of DLPC molecule from heme cofactor, is found to be correlated with opening/closing events of the other important channels of CYP1A2. This work thus shows that the dynamic of channels in P450 enzymes may also be influenced by the nature of phospholipids present in their vicinity.

In the same regard, Otyepka and his collaborators used a synergy of MOLE, the standard structure-based approach for identifying access channel, and a bias-exchange molecular metadynamics (BE-META) to study in silico the mechanism(s) of ligand passage through the flexible channels of CYP3A4 embedded in a 1,2-dioleoyl-sn-glycero-3-phosphocholine (DOPC) membrane environment [67]. The variation of the free energy for the passage of a caffeine metabolite through the channels was systematically looked at and several interesting facts emerge from this study. The free energy is systematically higher toward the mouth of the channel and systematically decreased along the channel when the channel-traveling molecule goes from the protein surface to the catalytic cavity. A CYP3A4 channel, namely 2af, seems to not permit exiting through it due to a very high free energy at its mouth for the passage of the product. Other channels, namely 4 and S, appear to be more prone to be exit channel for this caffeine metabolite, due to a considerably smaller value for the free energy of passage of the metabolite at their mouth. These results provide new insights into the mechanism of ligand release from CYP3A4. Again, the F/G region was found to play a prominent role in the observed channel malleability.

All of these results indicate that at least two notions have to be kept in mind when looking at channels and ligand selectivity in P450 enzymes. The irst is the F/G cassette of the P450 molecule that corresponds, from N to C termini, to the F- and F’-helices, the F’/G’ lop, and the G’- and G-helices. The second is the fact that the P450 molecules in eukaryotic cells are membranous. Bioinformatics models show that the hemoprotein is partly embedded in one layer of phospholipids. The association to the membrane and the F/G cassette are key elements in the relationships between channels and ligand selectivity in P450 enzymes. 

## 4. The F/G Region and Aromatic Residues

The F/G region of P450 enzymes have not only been highlighted by molecular dynamics simulations, but also by biochemical and biophysical experiments, and this, both for bacterial and eukaryotic P450s. Historically, a first sight at the importance of the F/G region was emerging from the comparison of the different crystal structures of CYP2B enzymes complexed with ligands of different sizes or in ligand-free conformation. It was clear that the F/G region of these enzymes appears to be the most impacted by ligand binding (See Figure 1 in [68]).

The F and G helices define the most common channels [38]. These two helices constitute a structural unit that participates to the distal surface of P450s. Whether a given channel opens up depends on the physicochemical properties of the interface between the F/G structural block and the rest of the P450 molecule, and also on structural features, such as the extent of the F/G loop and the arrangement of F’ and G’ helices within the F’/G’ region in mammalian P450 enzymes. Moreover, the F helix is observed to be broken in crystal structures of some P450s, such as CYP1A enzymes.

In molecular dynamic simulation of CYP2A6, the flexibility of the F’/G’ region is found to be much higher than that suggested by analyzing the B-factor of both the corresponding crystal structures. Moreover, the shift of the F-helix and of the F/F’ loop in CYP2C9 in high-temperature molecular dynamics simulations are shown to close the solvent channel and to open the channel 2b. [69].

A large conformational shift of the F/F’ loop is also seen in CYP3A4 crystal structures complexed with ketoconazole (a P450 inhibitor) or erythromycin (a CYP3A4 substrate) [70]. This local movement of the peptide backbone leads to a dramatic increase of the active site volume (of about 80% with erythromycin). A thorough looking at the motions of the different secondary structures forming the F/G region shows a distinctive flexible adaptation of this region to the type of ligand bound. With both erythromycin and ketoconazole, the F’/G’ cassette moves away in the same direction from its position seen in the ligand-free crystal structure. But with one type of ligand, the F and G helices moves simultaneously in the same direction relative to their positions in the ligand-free structure, but with another type of ligand they move in opposite direction. This result points out how Nature may use a few specific sequence motifs to modulate the channel opening of the different P450 enzymes, and thus their associated ligand specificity, by using local flexibility of some definite secondary structure elements.

This motion was observed both when comparing the crystal structures of the same P450 complexed with different ligands and in silicoby revealing the malleability of this region in P450 enzymes. However, the movements of the different elements forming the F/G region are not always easy to identify or to simulate in molecular dynamics, since, for instance, four of the 24 crystal structures of CYP3A4 available in the Protein Data Bank (PDB) have missing residues in the F and F’ helices. In this respect, it is interesting to note that two of the less-studied channels that were seen with CAVER in P450 enzymes, namely channels 3 and 4, have their opening right in-between the F and the G helices, one being very close to the F’/G’ helices(channel 4) and the other close to the F/F’ loop [38]. It has been shown that one of them, channel 4, couldbe predominantly a metabolite egress channel in CYP3A4 [67]. 

Molecular dynamics simulations pinpoint aromatic amino acid residues as being directly involved in the F/G region motions that control the opening and the closing of access channels. When applied to human CYP2A6, molecular dynamics simulations showed that one of its CAVER channels could be preferred for coumarin access to the active site. The opening of this channel, in particular, was shown to be dependent on the rotation of the side chain of a phenylalanine residue, Phe209. This result shows that channels may be opened by a rather simple mechanism, depending on the recognition of a ligand and an induced flip-flop type movement of an aromatic side chain [69].

A flattened cylinder is better adapted to describe the envelope of a P450 ligand molecule, which is most of the time of discoidal shape. In a recent study, Petitjean and his collaborators have analyzed 24 crystal structures of human CYP3A4 with their newly developed algorithm CCCPP (Computing Cavities, Channels, Pores and Pockets) [39] that finds out the different ways used by a cylinder to escape the catalytic cavity. They choose CYP3A4 because its numerous crystal structures are showing an impressive flexibility of some parts of the protein, suggesting that the ligand access and the exit routes might differ from a P450 structure to the next, and that these differences might be correlated to ligand physicochemical properties [71]. By doing so, they have classified the 24 crystal structures in three groups of different conformations, on closed, named C, in which a substrate-free CYP3A4 structure is found, and two open conformations, named O1 and O2. They have also identified a series of crucial physicochemical properties for a molecule to be a CYP3A4 ligand, namely its hydrophobicity (LogP), the flatness of the cylinder that describes it, its ability to П-П interactions, as measured by the Hückel П-energy, and the number of ligand molecules that can bind simultaneously to CYP3A4. In this interesting in silico study, they have highlighted the key role of two amino acid residues, Leu212 and Phe213, in triggering and controlling the shift between the three different conformations of CYP3A4. 

Phe213 has been also identified independently as a critical residue in allosteric regulations that were observed with CYP3A4 when two substrates, carbamazepine and progesterone, were mixed [72]. A strong activation of carbamazepine epoxidation and the simultaneous inhibition of progesterone hydroxylation was observed, and carbamazepine was found to preferentially bind to the catalytic cavity, whereas progesterone was shown to preferentially bind to the peripheral site that was observed on the crystal structure from which it plays its activating role on carbamazepine metabolism, a typical allosteric regulation. Phe213 was shown to be responsible for the observed synergetic effect of progesterone on carbamazepine epoxidation reaction, together with two other aromatic residues. 

By molecular dynamic simulations of human CYP3A4, it was predicted that the monooxygenation products of temazepam, which is a benzodiazepine hypnotic, and testosterone are each using a different channel for exiting the catalytic cavity once produced. Two phenylalanine residues were identified as a gating control for this differential channel opening that depends on the substrate structure. Molecular dynamics simulations suggested that the release of the bound product was controlled in part by aromatic side chains at the opening of the egress channels. Two phenylalanine residues were prominent in that control in CYP3A4, Phe215, and Phe220 [73].

In a recent study of the binding of metconazole, an enantiomeric P450 inhibitor, to CYP3A4, it was shown that two aromatic residues were involved in the differential binding of the RS and SR enantiomers within the catalytic cavity [74]. The chlorobenzene ring of the inhibitor interacts with Phe108 in the RS enantiomer bound structure and with both Phe108 and Phe304 in the SR enantiomer bound structure because of different positioning of the inhibitor. Besides these two active site residues, the peripheral residues Phe213 and Phe215 are positioned differently to each other in both of the structures. In the RS enantiomer structure, the phenyl side chains of Phe213 and Phe215 are positioned away from each other, whereas in the SR enantiomer structure, Phe213 and Phe215 are oriented toward each other. This again suggests that the different positioning of each enantiomer within CYP3A4 active site could result from each eniantomer using different access channels. 

It thus appears that P450 enzymes show structural regions with major differences in flexibility. Some peripheral regions are found to be very flexible, such as the F/G region, while the heme binding core of the P450 molecule is quite rigid. This organization accounts for the duality in P450-catalyzed reactions, to a wide variety of substrate metabolized (explained by the wide catalog of flexible structural arrangements at the protein surface), the dioxygen activation at the core heme iron ion is identical from a P450 to another (explained by the common rigidity of the core protein). Several questions remain unanswered, in particular, the crosstalk between the aromatic residues impacting the conformational shift and the binding of different ligands impacting the flexibility of those two key aromatic residues. Only experiments could provide information on this interesting topic.

## 5. The Membrane

Since eukaryotic cytochromes P450 are membrane-bound enzymes, to study the interplay between the membrane bilayer and P450 channels is of interest despite the limited number of such studies available. Initially proposed to be folded in eight transmembrane segments [75], microsomal P450s are now well known, for almost thirty years, to be anchored to the endoplasmic reticulum membrane through a N-terminal signal sequence that forms a helix that spans the membrane with the N-terminus in the lumen of the endoplasmic reticulum and the rest of the protein at the surface on the cytosolic side [76,77]. Two full-length microsomal P450s, namely CYP19 aromatase [78] and yeast CYP51 [79], have also been crystallized and their structure shows clearly the position of the N-terminal membrane anchor relative to the rest of the protein.

In a recent review, the different aspects of the possible effects of the membrane and its constituents on P450 functioning have been presented and discussed in great details, mostly originating from molecular dynamics simulations [80]. It is proposed that, when bound to the membrane, the eukaryotic P450 protein dips its F’/G’ region in the lipid bilayer [81,82]. The composition of the membrane may also affect the P450 functioning; and the orientation of the catalytic domain of the P450 enzyme relative to the membrane was shown to change depending on the charge of the bilayer phospholipids in molecular dynamics simulations [83].

Again, the role of molecular dynamics simulations was and still is preponderant. More than a decade ago, Wade and her collaborators did RAMD simulations on CYP2C5, a mammalian membrane-bound enzyme [84], which proposed aputative role for the membrane on differential channel opening and closing [85]. In this study, three snapshots from a trajectory in which a substrate molecule, 4-methyl-*N*-methyl-*N*-(2-phenylpyrazol-3-yl)benzenesulphonamide, exits from CYP2C5 catalytic cavity show that the F-G region is subjected to a conformational change that allows for a egress channel to open, thus allowing the substrate to exit. The atomic models of six human P450 enzymes including CYP3A4 anchored to a lipid bilayer were constructed and investigated by molecular dynamics simulations for the position and orientation of the P450 protein on the model membrane. All of the studied P450s were predicted to be partially immersed in the lipid bilayer, whereas the N-terminal part and F’/G’ loop are deeply immersed. The proximal side of the enzyme faces the cytosol, whereas the distal side, where openings of substrate access and product release channels to the active site are primarily located, points toward the lipid bilayer. Access channels with openings in the vicinity of the B/C and F/G loops are typically positioned below the phospholipid head groups, whereas the solvent channel points toward the membrane–water interface. This orientation of the P450 in the membrane suggests that membrane-anchored CYPs have evolutionarily adapted to facilitate the uptake of nonpolar substrates from the membrane and the release of polar products to the membrane–water interface [82].

The structure of human CYP2C9 is known from X-ray experiments, but its mode of insertion into the membrane remains unsolved [86]. Atomistic models of complete CYP2C9 embedded in a bilayer membrane were constructed and evolved by molecular dynamics simulations [81,87]. The entry of the substrate access channel was found to be facing the membrane interior, while the exit of the product egress channel is found to be located above the interface between phospholipid heads and the water solvent.Moreover, the positions of the mouths of the substrate access and the product egress channels roughly correspond to the positions of energy minima for the location of CYP2C9 substrate and its metabolite in the membrane [87]. The results that are deduced from these in silico studies also agree well with the few known experimental data about the membrane positioning of cytochromes P450.

An important point to keep in mind is the presence of lipid rafts, or membrane ordered microdomains well known in eukaryotic cells, both in animals and plants [88,89,90]. The P450 enzymes seem to not have the same affinity for these cholesterol-rich microdomains. Using wild-type and artificial chimeras between human CYP1A1 and human CYP1A2, conclusive evidence were presented showing that, despite a 82% sequence identity between them, these two human P450 enzymes have a totally different affinity for the lipid rafts in mammalian cells [91]. The preferential association of the P450 molecules with the bilayer either in non-raft areas or in rafts should most probably influence somehow the dynamics of the channels, and hence, their range of ligand selectivity. A molecular dynamics study of CYP3A4 embedded in bilayer environments presenting increased levels of cholesterol (from 0% up to 50%) shows that, as cholesterol amounts increase, the immersion an inclination of the CYP3A4 catalytic domain change relatively to the membrane. moreover, the pattern of CYP3A4 accessible channels is altered [92]. The physiological impact of this findings on P450 functioning related to cholesterol homeostasis and lipid raft microdomains remains to be precised.

## 6. Experimental Validation of Channels in Cytochromes P450

Molecular dynamics simulations analyze the channels connecting the buried active site to the bulk solvent in a dynamic view, and, in this regard, give invaluable and complementary information on the system. The accessibility of the occluded catalytic cavity of P450 enzymes originates from the analyses of crystal structures, which offer only a static view, and it is known that shortcomings, such as crystal packing causing non-native contacts and constraints, may affect conclusions that are deduced from the comparison of crystal structures [93].

With this in mind, it appears that any experimental validation of the channels in P450 functioning could be of great importance to consider that in silico predicted channels are not bias artifacts. Historically, the first validation of the role of a channel in the function of a P450 was obtained some twelve years ago with insect CYP6B1. This P450 metabolizes furanocoumarins, and a mutant Ile115leu was obtained and analyzed that has a profound influence on the furanocoumarin selectivity of CYP6B1. It was shown, on model structures, that this mutation is located close to the edge of the heme coenzyme. It was further shown that this residue forms a constriction in a product egress channel of CYP6B1. The mutation was modifying this constriction, and thus, the property of the channel in a manner coherent with the observed biochemical data [94].

Hollenberg and his coworkers have identified several putative access channels in CYP2B1 enzyme using CAVER [95]. They constructed a site-directed double mutant, Tyr309Cys Ser360Cys, in which a disulfide bridge was introduced inside one of the multiple CAVER channels, thus obstructing it. The wild-type parental enzyme catalyzes both benzphetamine *N*-demethylation reaction and *O*-deethylation reaction on a 7-ethoxy derivative of coumarin. The variant with the disulfide bridge was found to metabolize benzphetamine at the same rate as the wild-type enzyme, the double mutation was not affecting this activity. However, this variant was catalyzing the O-deethylation of the coumarin substrate at a rate that was five-fold lower than that of the wild-type CYP2B1. The differential modification of the dynamics of the multiple access channels in CYP2B1 by blocking one of them had resulted in a differential impact on substrate specificity by filling the empty space in the protein periphery. This empty space was found to be part of channel by in silico algorithms. It can be deduced that, most probably, channels are implied in the substrate preferencein that case. This important work was also showing that different substrates may use different channels to access the active site in P450 enzymes. This experimental result confirmed what was deduced some fifteen years ago from molecular dynamics simulations on three bacterial P450s suggesting that the channel opening mechanisms are adjusted to the physico-chemical properties of the substrate and can kinetically modulate protein-substrate specificity [96]. This opens the way to a study of the relationships between the functions and the composition of the channel networks, as they are systematically observed in P450 enzymes.

**Figure 4:**
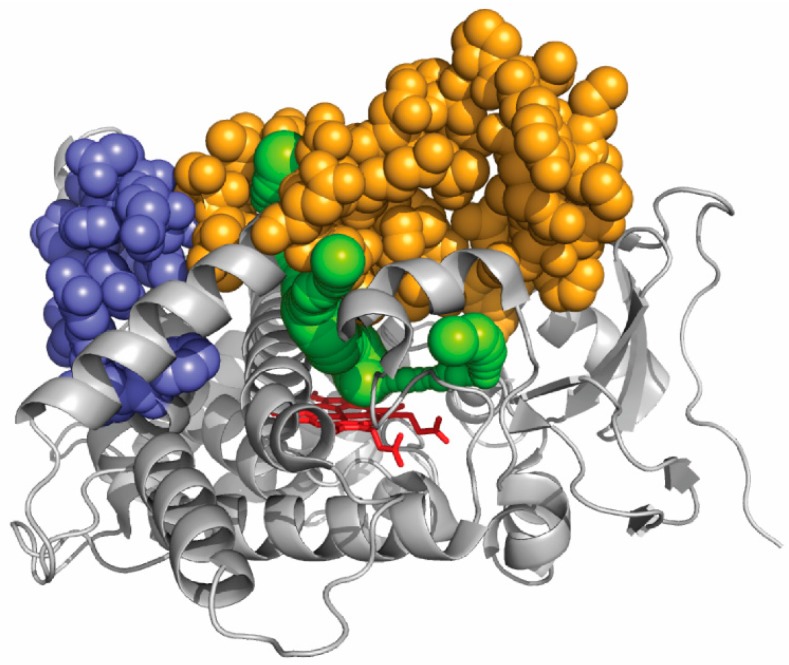
Visualization on CYP1A1 crystal structure of the regions mutated and the ligand access channels. The protein structure is represented as cartoon with the heme represented as red sticks. CAVER channels are in green. The sequence segment that, when mutated, shows the same polycyclic aromatic hydrocarbon dual specificity as wild-type CYP1A enzymes is in purple. The sequence segment that, when mutated, shows an impact on the substrate specificity for hydrocarbon substrates is in orange. This later segment contacts the different CAVER channels. The key sequence elements that were identified in this work are precised in Urban et al. [100].

The active site of CYP1A enzymes, as seen in a crystal structure, is sufficiently large to easily accommodate any 5-ring polycyclic molecule [97,98]. From benzo[*a*]pyrene, CYP1A1 produces 4,5-, 7,8-, and 9,10-epoxides in significant amounts [99]. This reveals that the orientation of benzopyrene in the active site seems fairly flexible. An unanticipated result was recently reported for CYP1A enzymes that were shown to strongly differentiate polycyclic aromatic hydrocarbons with two main classes of specificity, identified as two main global mechanisms of action, on the basis of their size [100]. Multiple mutations affecting a surface region of the protein far from any exit of a CAVER channel in CYP1A enzymes, but not impacting enzyme activity appeared to have no or limited impact on the dual polycyclic substrate size discrimination (Figure 4). In these mutants, as in wild-type CYP1A enzymes, the discrimination between small and large substrates appears to stem fromtwo very different global kinetic behaviors. However, when a region of the protein surface that correspond to an exit of several CAVER channels was multiply mutated, drastic changes in this size global discrimination were observed. These experimental results, based on kinetics, correlate well with the channels identified in silico. This suggests that access channels in CYP1A enzymesindeed influence substrate specificity for polycyclic aromatic hydrocarbons by modulating the access to the active site on the basis of a primary recognition of the substrate size. It can also be deduced from what precedes that at least part of substrate determinants of the observed size selection between small and large polycyclic substrates does not lie in the active site structure.

One of the channels exits between the F- and G-helices and is lined by a break in the F-helix, which is a feature characteristic of CYP1 structures. The three-residue disruption of helix F in CYP1A2 is extended to five residues in CYP1A1. This suggests that this longer break in helix F and a slightly longer helix B′ are the only secondary structural differences that are observed between CYP1A1 and CYP1A2. Moreover, the tilt that was observed in X-ray structures between the N- and C-terminal moieties of helix Fis more pronounced in CYP1A2 than in CYP1A1. This causes the mouth of one of the four channels, namely channel 3, to be smaller in CYP1A2 than in CYP1A1. The break in helix F could thus be involved to a certain degree in the control of the substrate size specificity observed in this study. A combination between the substrate access channel and the F-helix break could act as a structural determinant of substrate specificity in this family of P450 enzymes. 

Similarly, mutation Phe240Ala in rat CYP1A1 has changed the selectivity of hydroxylation towards 2,3,7,8-tetrachloro-dibenzo-p-dioxin (TCDD) and 3,3′,4,4′-tetrachlorobiphenyl (PCB77) [101]. The F240A mutant presents an activity towards TCDD, whereas the wild-type rat CYP1A1 has no activity detectable for TCDD. On the opposite, the turnover number for PCB77 was greatly reduced in F240A mutant when compared to the wild-type enzyme. The molecular dynamics simulation performed on the wild-type and F240A mutant of rat CYP1A1, showed that the preferred channels (those that are open most of the time and are wider at bottleneck during the duration of the simulations) are not the same between the mutant and the wild-type P450 enzyme. The aromatic Phe240 residue was suggested to act as a gatekeeper for the movement of molecules within the channel 2b/2af. 

A natural genetic variant of CYP1A2, namely CYP1A2*11, was characterized for its activity towards two marker substrates, phenacetin and 7-ethoxy-resorufin, and was shown to be more impacted for phenacetin *O*-deethylase activity (10% of that of wild-type CYP1A2) than for ethoxyresorufin *O*-deethylase activity (30% of that of wild-type CYP1A2) [102]. This variant results from a single amino acid mutation at position 186, the wild-type Phe is mutated to a Leu residue. The impacted position is located at the periphery of the protein molecule and quite far from the active site. In the crystal structure, the alpha-carbon of Phe186 is distant of about 28 Å from the heme iron ion. In a recent work, the aromatic residue 186 was proposed, based on molecular simulation studies, to exist in two conformational states, corresponding to the open or closed conformation of one of the several access channels of CYP1A2 network [103]. This indicates that Phe186 could act as a gating residue.

One of the structural determinants of substrate specificity for cyclophosphamide, which is an antitumor prodrug, has been recently identified in CYP2B6 and CYP2B11 enzymes. CYP2B11 presents a K_M_ for cyclophosphamide 50-fold lower than that of CYP2B6 [104]. The CYP2B6 sequence was sliced into 15 contiguous sequence modules and each of them was used to replace its counterpart into the CYP2B11 backbone, generating 15 individual chimeras. One of the 15 chimeras, namely, chimera G, was found to be exhibit an affinity for cyclophosphamide that was similar to that of CYP2B6 though its sequence is that of CYP2B11 with a minor change of six residues into those of the CYP2B6 sequence. When positioned on the crystal structure of CYP2B6, these changes are found to be located on the F’/G’ region, inside the F/G region. The entrance of channel 2f, one of the several access channels viewed in CYP2B6, is lined by the F’/G’ elements. When these fifteen CYP2B6/2B11 chimeras were assayed with a marker CYP2B substrate, namely 7-ethoxy-4-trifluorometyl-coumarin, they all exhibit a similar affinity for this fluorogenic substrate. Both substrates seem thus to use different access routes to the catalytic cavity as was suggested in a previous study [105]. The structural changes in chimera G that strongly impact the specificity for cyclophosphamide in CYP2B11 are not impacting the coumarin derivative, indicating that coumarin may compensate the effect of the sequence swap that affects channel 2f, by using another channel. This alternate route would not be accessible to cyclophosphamide. This work thus suggests that the coumarin derivative may use several access channels in CYP2B enzymes, whereas cyclophosphamide would only use channel 2f. In other study on CYP2B enzymes, it was shown that a concerted movement of helices F through G seems to facilitate an enthalpically driven binding of ligands of various sizes without perturbing the P450 core where the catalytic site lies [17,106]. 

**Figure 5:**
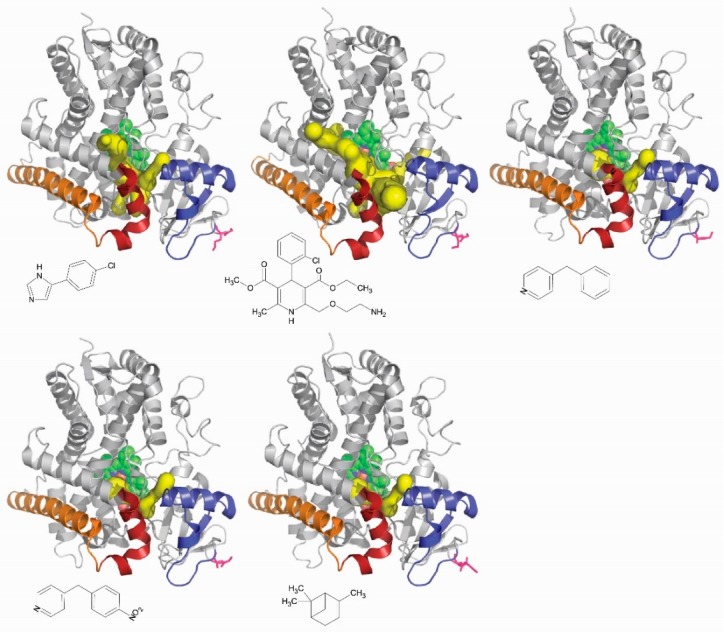
The crystal structures of CYP2B6 (From left to right and from the top to the bottom: 3IBD, 3UA5, 3QOA, 3QU8, and 4I91) bound to various ligands are shown with the structure elements important for cyclophosphamide affinity colored in blue, dark red, and orange. The ligand complexed is represented under each of crystal structures. The two N-terminus residues of each structure are depicted as red sticks. The heme is represented as green van der Waals spheres. Predicted access channels (depicted as yellow surfaces) were calculated using the CAVER 3.0.1 plug-in of PyMOL. For the same P450 enzyme, the combination of channels accessible changes from one ligand to the other. The role of each structure elements highlighted in cyclophosphamide affinity is explained in details in Lautier et al. [104].

The network of channels observed in all P450 enzymes is thus likely involved in the control of their substrate specificity. Different ligands of the same P450 could reach the active site through different channels. It is known that the network of channels seen for the same P450 is different from one complex to the other depending on the ligand bound (Figure 5). Channels are an element of the structural determinants of the substrate specificity in P450 enzymes. Another enzyme system, different of cytochromes P450, was tested for that and was found to be positively answering. Mutations of amino acid residues lining the access channels of haloalkane dehalogenase altered the substrate specificity of the respective enzymes. The authors have identified all of the channels in the network that this enzyme, which is not a cytochrome P450, exhibits. In one of the channel, they identify key amino acid residue lining the channel and subjected them to selective molecular directed evolution. They isolated a DhaA variant that has five of the positions in this channel modified relatively to the wild-type enzyme. These are Ile135Phe, Cys176Tyr, Val245Phe, Leu246Ile, and Tyr273Phe. This variant enzyme presents a 32-fold higher activity with trichloropropane, a recalcitrant anthropogenically generated pollutant, than the wild-type enzyme. The K_M_ for trichloropropane was found to be the same for both the variants and the wild-type enzymes [107]. Engineering an access channel may thus result in a profound modification of the catalytic activity of an enzyme, thereby showing that in silico found access channels are indeed playing a role in enzyme function.

## 7. Conclusions

The first X-ray crystallography work on P450s was reported by Poulos and his associates, and described the structure of camphor-bound P450 101A1 [108,109], then the crystal structure of the ligand-free enzyme [44], raising questions about how the substrate gains access to the active site, which was described by Poulos as “inaccessible to the outside world” [110]. This question has been addressed since by a combination of experiments in vitro and of molecular dynamics simulation in silico. The common image is a combination of access channels forming a flexible network of channels connecting the heme iron to the distal surface of the P450 protein. The key structural element found in all P450s tested is the F/G region, also observed to be crucial for P450 function in a series of biochemical experiments. Another key structural element is a group of aromatic residues playing an important role in the control of closing-opening of the access channels. These channels are involved in a first, global recognition of the ligand at their entrance on the protein surface before the ligand reaches the catalytic cavity. This global recognition of the ligand by channels is also influenced, in some cases, by the associated redox protein [111,112]. Moreover, different channels may exhibit different ligand specificity. In other words, two different ligands may access the catalytic cavity of the same P450 by using different route. 

After identifying the access and egress channels in P450 structures, the different in silico approaches now precise the physicochemical properties of these channels and also indicate how the composition of the membrane or the type of the bound ligand may affectthe network of accessible channels. However, there is still a gap between these in silico findings and what so-called wet biology shows. The filling of this gap appears to us as one of the most urgent prospects in this field of P450 biochemistry.

Future prospects on channels in P450 enzymes could include the possibility to transfer a specific channel from a P450 to another, in which this specific channel is missing. The mutant enzyme thus constructed could be assayed with a series of substrates in order to assess if and how the specificity was modified by the insertion of the new channel. Such an experiment would be quite uneasy to carry out because several specific residues should be simultaneously mutated, but its results would be most valuable to precisely determine how channels do control ligand specificity. 

Another interesting prospect is the study of the potential implication of channels in the control of P450 uncoupling, i.e., the ratio between productive and abortive catalytic cycles. Such a study would lead to a better knowledge of what combination of amino acid residues in a P450 enzyme distinguishes a ligand (a bound molecule that not reaches easily the productive step of the catalytic cycle) from a substrate (a bound molecule that reaches the productive step). All of these future studies will rely on extensive and specific multiple mutations in P450 enzymes, and, for the moment, their feasibility and outcome are hardly predictable.

## Abbreviations

CCCPP	Computing cavities, channels, pores and pockets
P450	Cytochrome P450
QSAR	Quantitative structure-activity relationships
RAMD	Random acceleration molecular dynamics
REMD	Random expulsion molecular dynamics
